# An imported case of Bundibugyo virus infection, France, June 2026

**DOI:** 10.2807/1560-7917.ES.2026.31.31.2600627

**Published:** 2026-08-06

**Authors:** Morgane Mailhe, Delphine Pannetier, Estelle Yamani, Lyderic Aubert, Arnaud Tarantola, the Ebola Investigation Team, The Ebola Investigation Team, Sylvain Baize, Hervé Blanchard, Emilie Daimant, Diane Descamps, Julie Figoni, Adina Henegar, Solen Kerneis, Quentin Le Hingrat, François-Xavier Lescure, Camille Peneau, Nicolas Roux

**Affiliations:** 1 Infectious disease department, Hôpital Bichat-Claude Bernard, Assistance Publique – Hôpitaux de Paris Nord Paris, France; 2 National Reference Center for Viral Hemorrhagic Fevers, Laboratoire P4 Jean Mérieux INSERM, Lyon, France; 3 Agence Régionale de Santé Île-de-France, Saint-Denis, France; 4 Public Health Emergency Operations Centre (CORRUSS), Ministry of Health, Paris, France; 5 Direction des Régions, Santé publique France, Saint-Maurice, France; 6 The members of the Ebola Investigation Team are listed under Collaborators

**Keywords:** Ebola, imported infection, epidemiology, blood-borne pathogens, filoviridae

## Abstract

In June 2026, an intensive care physician deployed in the Democratic Republic of the Congo and previously vaccinated against Ebola virus developed fatigue, nausea and headaches while returning to France. Bundibugyo virus was identified with RT-PCR. The patient was treated with remdesivir and recovered fully. Contact tracing identified five low-risk contacts and no secondary cases. Stringent infection prevention and control measures and multidisciplinary collaboration are important when managing suspected or confirmed Ebola disease cases, even with low viral loads.

Ebola virus (EBOV) was first detected in former Zaïre, current Democratic Republic of the Congo (DRC), in 1976 [1]. Since then, outbreaks have been caused by several orthoebolaviruses, including Bundibugyo virus (BDBV), which was identified in 2007 in Uganda [2,3]. An intense epidemic caused by BDBV is currently ongoing in the DRC province of Ituri and neighbouring areas of Uganda [4]. Bundibugyo virus is considered somewhat less lethal than other orthoebolaviruses, but rapid identification, adequate patient management and isolation is key to preventing secondary cases [5].

## Case description

Patient A, an intensive care physician in his fifties with more than 10 years of clinical and field experience of working with a medical nongovernmental organisation, had been deployed in an Ebola treatment centre in Ituri since 15 May. Patient A had no previous medical history, was fully vaccinated against EBOV in 2019 and reported using appropriate personal protective equipment (PPE) during the ongoing epidemic when caring for hundreds of suspected or confirmed BDBV cases.

On Friday, 19 June, Patient A experienced profound fatigue with no other symptoms, which he attributed to his intense 5-week mission, prompting his decision to return to France for rest and recuperation. He travelled on the same day from Bunia, Ituri, to Kinshasa on the only available daily, 5-hour United Nations (UN) flight. Patient A was screened on departure and arrival but remained afebrile. He did not use the restrooms on board and spent the weekend in a single room of a guesthouse with no close contact with other persons. On 20 June, Patient A developed a headache which he attributed to moderate alcohol consumption the previous evening as he rarely drinks. On Monday 22 June, he travelled with an 8-hour commercial flight from Kinshasa to Paris, arriving in Paris on Tuesday 23 June.

During the flight, Patient A experienced increasing fatigue, dizziness, nausea, abdominal pain, recurring headaches requiring paracetamol self-treatment but no fever, vomiting, diarrhoea or bleeding. He did not use the restrooms on board, had no contact with other passengers and no prolonged contact with the crew. Fully aware that these symptoms could indicate orthoebolavirus infection, Patient A placed himself on a far end of the airport shuttle train in Paris, distancing from other passengers, before voluntarily self-presenting to the Charles de Gaulle Airport Medical Center (Service Médical d’Urgence, SMU) and informing the staff about his profession and recent field mission to Ituri. The SMU team had no direct physical contact with Patient A and immediately placed him in an isolated room.

On 23 June, the SMU notified the Greater Paris Area high consequence infectious disease (HCID) reference health centre team and national procedures were initiated. The Regional Health Agency (ARS) and the regional field office (CR) of Santé publique France (SpF, the French National Public Health Agency) for Île-de-France (Paris and seven neighbouring departments) were notified. A multidisciplinary meeting with the HCID, the ARS, the SpF CR regional team, the physician of the Charles de Gaulle Airport, the Assistance-Publique – Hôpitaux de Paris, a SAMU emergency mobile resuscitation team, the infection control team and the National Reference Centre for viral haemorrhagic fevers (NCR-VHF) was held the same day at 1:30 pm. Patient A was considered as a probable BDBV case based on the symptoms and the anamnestic information, despite no identified exposure.

Given the nausea becoming more intense and with the risk of vomiting, Patient A was transferred from the airport to Hospital A via SAMU using an EpiShuttle. He was hospitalised in a negative pressure room at the HCID unit on the evening of 23 June. He received symptomatic care, and intravenous remdesivir was started on 25 June for a 10-day course, in accordance with the remdesivir arm of the PARTNERS trial [6]. Furthermore, a dose of monoclonal MBP-134 antibody was obtained from the manufacturer on 27 June but proved unnecessary.

Patient A remained afebrile throughout his hospital stay but experienced a few episodes of mild diarrhoea. The symptoms decreased as early as 26 June, and the patient was discharged after 11 days in hospital.

## Laboratory analyses

On 23 June, at the virology laboratory of the treating hospital, a blood sample of Patient A was tested with BioFire Global Fever Special Pathogens Panel PCR assay (BioFire Defence, Salt Lake City, the United States). Test results ruled out dengue viruses and *Plasmodium* spp. as causative agents, returning positive for orthoebolavirus (quantification cycle (Cq):  37). However, the RealStar Filovirus Screen RT-PCR Kit 1.0 (Altona Diagnostics GmbH, Hamburg, Germany) yielded a negative result for orthoebolaviruses. Later that night, BDBV RNA was detected in the patient’s plasma at the NRC-VHF with an in-house, specific BDBV RT-PCR test. The patient was considered confirmed, pending retesting. A new series of RT-PCR test runs on 24 June showed a low viral load, close to the detection limit of the assay. On 26 June, sequencing of amplicons at the NRC-VHF confirmed the presence of BDBV RNA in the patient’s plasma sample. The sequences were obtained from the RT-PCR amplicons and therefore were not deposited in a database because of their very short lengths.

## Public health measures

The Public Health Emergency Operations Centre (CORRUSS) of France’s Ministry of Health was mobilised from the evening of 23 June to trace contacts on the flights while the ARS/CR interviewed the SMU on 24 June. On 24 June, the HCID clinician interviewed the patient on all possible exposures using the SpF questionnaire and completed the written notification. Patient A was once again carefully interviewed by ARS to determine types and times on symptoms onset, and contacts with other people. The ARS and SpF CR teams documented events at SMU to identify potential contacts.

Given the mild clinical course and the patient’s low viral load even 4 days after symptom onset, the contact groups were classified according to RAGIDA recommendations [7,8]. The investigators did not have access to the passenger seating plan of the UN flight in DRC. Crew members, SMU and SAMU staff were considered non-contacts. Five passengers seated in the eight seats closest to Patient A were considered as low-risk contacts: a foreign national in Belgium travelling to Lebanon; a Dutch resident in the Netherlands; two French residents, one in the Occitanie *Région* and one in Île-de-France, the latter returning to Kinshasa for a brief stay; one foreign national returning to Brazzaville later that week, currently staying in Île-de-France. Other passengers on the commercial flight were considered non-contacts, and no high-risk contacts were identified. The national focal points for high-risk zoonotic and emerging diseases at SpF informed their counterparts in Belgium and the Netherlands, and a message was forwarded through the EpiPulse network of the European Centre for Disease Prevention and Control (ECDC) and reported via the Early Warning and Response System (EWRS) of the European Commission.

The SpF’s Ebola response protocol specifies no measures for non-contacts, and self-monitoring with free movement and daily ARS phone calls for low-risk contacts. The ARS initiated contact tracing and daily telephone monitoring on 24 June. At the time of publication, no contact had developed any clinical symptoms. A careful and comprehensive review of procedures and personal protection equipment with the convalescent patient identified no breach in procedures or accidental exposure to blood or body fluids.

## Discussion

We describe here an imported case of BDBV infection in a healthcare worker with no secondary cases. Other cases of BDBV acquired in the current epidemic were medically evacuated to Germany with no secondary cases [9,10]. Fever is present only in around 75% of patients with BDBV infection [11]. This estimated proportion of fever is, however, likely overestimated as fever is one of the clinical criteria for testing for orthoebolaviruses. Also, as the incubation period may vary (range: 2–21 days), temperature screening will therefore not be a reliable method for detecting imported cases.

Ebola virus is known to be transmitted through blood and semen [12]. Although EBOV RNA can be detected in various body fluids [13] such as vomitus, stools or sweat, the infectivity of these fluids, when not mixed with blood, remains unclear. In our investigation, we did not identify any accidental exposure to blood or body fluids or no breach in prevention and control measures, thus the source of the infection remains unidentified. Inability to identify transmission routes for healthcare workers with EBOV infection has previously been described [14]. Humidity and perspiration could facilitate EBOV in penetrating through protective material, but in experimental studies performed in high-humidity settings hours were required for EBOV to penetrate PPE material [15]. When properly applied, PPE remains effective protection, especially when changed routinely and when training is refreshed or repeated to prevent transmission [16,17]. As EBOV is present in oral fluids [18], the virus can persist in aerosols in experimental conditions [19] and may have been transmitted through respiratory droplets among nonhuman primates in a breeding facility [20,21]. Results from experimental studies in animals [22] and extensive field experience, however, indicate that respiratory transmission of EBOV between humans is rare and that transmission requires direct contact [12,23-25]. Irrespective of the quantities of EBOV RNA in respiratory droplets once symptoms have set in, experts consider that there is no risk of EBOV transmission before symptom onset [26].

## Conclusion

This event, involving a confirmed BDBV infection in a physician returning from DRC, had been anticipated by the health authorities. Few low-risk contacts and no high-risk contacts were identified, thanks to the patient’s awareness, adherence to preventive measures, nonjudgmental management in well-equipped conditions by trained staff and the robustness of established procedures and circuits. This reinforces the importance of stringent adherence to infection prevention and control measures. Humane, comprehensive and evidence-based management of EBOV or other HCID imported infectious diseases cases will help reduce the risk of undetected repatriation as was the case in 2007 [27], and its associated risks of secondary transmission.

## Data Availability

All data generated or analysed in this study are included in this published article.

## Members of the Ebola Investigation Team with affiliations

Biology of Emerging Viral Infections unit, National Reference Center for Viral Hemorrhagic Fevers, Institut Pasteur, Lyon: Sylvain Baize; Cpias, Saint-Denis: Hervé Blanchard; Agence Régionale de Santé Île-de-France, Saint-Denis: Adina Henegar, Camille Peneau, Emilie Daimant; Assistance Publique – Hôpitaux de Paris Nord, Hôpital Bichat-Claude Bernard: Diane Descamps, Solen Kerneis, Quentin Le Hingrat, François-Xavier Lescure; Direction des Maladies Infectieuses, Santé publique France, Saint-Maurice: Julie Figoni; Agence Régionale de Santé Occitanie, Toulouse: Nicolas Roux.
